# *Planococcus terreus* sp. nov. and *Planococcus flavus* sp. nov., Two New Species Isolated from Soil of the Napahai Plateau Wetland

**DOI:** 10.3390/microorganisms14092119

**Published:** 2026-09-21

**Authors:** Weidong Lu, Xingfeng Qian, Zheng Zhang, Zhiying Li, Zefen Yu, Yunxia Tian, Li Li, Yongxia Wang

**Affiliations:** 1 Yunnan Institute of Microbiology, School of Life Sciences, Yunnan University, Kunming 650091, China15284518344@163.com (X.Q.); 17860729198@163.com (Z.Z.); zyli@ynu.edu.cn (Z.L.); 2 State Key Laboratory for Conservation and Utilization of Bio-Resources in Yunnan, Yunnan University, Kunming 650504, China; zfyu@ynu.edu.cn; 3 Kunming Academy of Agricultural Sciences, Kunming 650034, China; 4 Xinjiang Key Laboratory of Biodiversity Conservation and Application in Arid Lands, Xinjiang Institute of Ecology and Geography, Chinese Academy of Sciences, Urumqi 830011, China

**Keywords:** *Planococcus*, polyphasic taxonomy, genome analysis, Napahai Plateau wetland

## Abstract

Two novel bacterial strains, designated YIM B11945^T^ and YIM B11997^T^, were isolated from soil samples collected in the Napahai Plateau wetland, Yunnan Province, China. The cells were aerobic, Gram-stain-positive, motile and non-spore-forming rods. The strains YIM B11945^T^ and YIM B11997^T^ showed the highest 16S rRNA sequence similarity with *Planococcus shixiaomingii* N022^T^ (97.5%) and *Planococcus koreensis* JG07^T^ (98.3%), respectively. The phylogenomic analyses based on the core genome sequences placed the two strains within the genus genus *Planococcus*. The average nucleotide identity and the digital DNA–DNA hybridization values between the two novel strains and their closest relatives were below the species delineation thresholds of 95% and 70%, respectively. The strains YIM B11945^T^ and YIM B11997^T^ possessed major fatty acids, which were anteiso-C_15:0_ and C_16:1ω7c_ alcohol, and anteiso-C_15:0_, iso-C_14:0_, C_16:1ω7c_ alcohol and iso-C_16:0_, respectively. The major polar lipids were phosphatidylglycerol, phosphatidylethanolamine, and diphosphatidylglycerol, and the respiratory quinones detected were menaquinone-7 and menaquinone-8. The genomic DNA G+C contents of YIM B11945^T^ and YIM B11997^T^ were 45.0% and 49.2%, respectively. Based on the phenotypic, genotypic and phylogenetic analyses, the strains YIM B11945^T^ and YIM B11997^T^ are considered to represent two new species of the genus *Planococcus*, for which the names *Planococcus terreus* sp. nov. and *Planococcus flavus* sp. nov. are proposed, respectively. The type strains are YIM B11945^T^ (=CGMCC 1.64737^T^=KCTC 43683^T^) and YIM B11997^T^ (=KCTC 43680^T^=NBRC 116630^T^).

## 1. Introduction

The genus, *Planococcus* belonging to the family *Caryophanaceae* and class *Bacilli* [1], was first established by Migula; the first identified species was *Planococcus* citreus, isolated from sea water [2]. Subsequently, the genus description was amended by Yoon et al. in 2010 and Gupta and Patel in 2020 [3,4]. At present, the genus *Planococcus* has a total of 34 species that have validly published names in the List of Prokaryotic Names with Standing in Nomenclature (https://lpsn.dsmz.de/genus/Planococcus, accessed on 1 January 2026) [5]. The members of the genus *Planococcus* exhibit Gram-positive or Gram-variable staining, are aerobic and non-spore-forming, have cell shapes including cocci or short rods, and are generally motile [4]. The genus *Planococcus* is widely distributed in different regions and environments, including marine [3,6,7,8,9,10,11] and high salt areas [12,13,14,15,16,17,18].

The Napahai Plateau wetland, located in the northwest of Yunnan Province, China, is a unique seasonal plateau wetland characterized by its low latitude and high altitude within the Jinsha River basin. It was designated as a Wetland of International Importance in 2004. This wetland is notable for its high microbial biodiversity and active microbial community [19], with microorganisms playing a crucial role in maintaining the energy balance. Therefore, it is essential to conduct research on the microbial diversity in the Napahai Plateau wetland. In this study, we clarify the taxonomic position of two strains, YIM B11945^T^ and YIM B11997^T^, isolated from wetland soil using a polyphasic approach. Our results indicate that the two strains represent two novel species of the genus *Planococcus*, proposed here as *Planococcus terreus* sp. nov. and *Planococcus flavus* sp. nov.

## 2. Materials and Methods

### 2.1. Isolation and Maintenance of the Organisms

Soil samples were collected within the area of E99°37′18″–E99°39′49″, N27°52′56″–N27°53′8″ in the Napahai Plateau wetland in June 2022. We attempted to cultivate numerically abundant species using a variety of different strategies (enrichment culture and variety media). Strains YIM B11945^T^ and YIM B119997^T^ were isolated from soil samples of Napahai Plateau wetland, located in Shangri La County of Yunnan province, southwestern China. To isolate strains, the soil sample was serially diluted in sterile river water, and 100 μL of the diluted solution was spread on International Streptomyces Project 2 agar plates (ISP2; BD). The inoculated plates were aerobically incubated at 28 °C for 3–5 days. After incubation, a distinct single colony was picked and purified by repeated streaking on ISP2 agar plates. Pure cultures were maintained on ISP2 agar and maintained at −80 °C in ISP2 broth supplemented with 20% (v/v) glycerol.

### 2.2. Morphological, Physiological, and Biochemical Characteristics

Growth was assessed at 28 °C for 3 days on several agar media: International Streptomyces Project 2 agar (ISP2; BD), Luria–Bertani agar (LB; BD), nutrient agar (NA; BD), tryptic soy agar (TSA; BD), and Reasoner’s 2A agar (R2A; BD). Gram staining was carried out as described by Magee et al. [20] and was confirmed by the 3% KOH lysis test [21]. Cell shape, motility and endospore production were observed by using light microscopy (BH-2, Olympus, Tokyo, Japan) and transmission electron microscopy (TEM; JEM-2100, Tokyo, Japan). Growth at various temperatures (4, 10, 15, 20, 25, 28, 30, 35, 40 and 45 °C) and pH ranges (4.0–11.0, at intervals of 1 pH unit) were determined on NA. Salt tolerance was tested on ISP2 medium supplemented with various concentrations of NaCl (0–10%, at intervals of 1.0%, w/v). Growth under anaerobic conditions was determined after incubation in an anaerobic jar (GasPak Anaerobic systems, BBL, Franklin Lakes, NJ, USA) on ISP2. Catalase and oxidase activities were determined using 3% (v/v) hydrogen peroxide and Kovac reagent [22], respectively. The evaluation of glucose oxidation and fermentation, as well as hydrolysis of starch, gelatin, casein, and Tween 20, 40 and 80 was carried out based on the method described by lányí [23] and Smibert and Krieg [24]. Nitrate reduction was assessed in ISP2 broth containing 0.2% KNO_3_. Nitrite was monitored with naphthylamine/sulphanilic acid reagents, while residual nitrate was monitored with the aid of Zn powder; gas production was detected in Durham tubes. Detection of H_2_S was done using ISP2 liquid medium containing 0.01% (w/v) of L-cysteine; a strip of paper impregnated with lead acetate in the neck of the tube acted as an indicator. Indole was produced in ISP2 liquid medium containing 0.5% (w/v) tryptone detected with p-dimethylaminobenzaldehyde. Acid production from carbohydrates was determined using an API 50CH system (bioMérieux, Lyon, France), and additional enzyme activities were analyzed using API ZYM and API 20NE kits (bioMérieux), according to the manufacturers′ instructions.

### 2.3. Chemotaxonomic Analysis

Strains YIM B11945^T^ and YIM B11997^T^ were cultured until exponential growth under optimum growth conditions, harvested by centrifuging and subjected to chemical classification. The strains’ cellular fatty acid profiles were examined as described by Sasser [25] and the fatty acid methyl esters were prepared and analyzed through gas chromatography (model 7890, Agilent, Santa Clara, California, USA) and the Sherlock Microbial Identification system (MIDI, version 6.0; MIDI database: TSBA6). Respiratory quinones were extracted according to the method by Collins et al. [26] and analyzed using HPLC [27]. The extraction of polar lipids was done using a chloroform–methanol solvent system and analysis by TLC (thin-layer chromatography) on silica gel 60 F254 thin-layer plates (Merck, Darmstadt, Germany) [28]. Two-dimensional chromatography was used for the separation of the separate elements, and identification was done by their *R_F_* values together with their reactions to specific staining reagents [29,30].

### 2.4. 16S rRNA Gene Sequencing and Phylogenetic Analysis

Genomic DNA extraction, PCR amplification of the 16S rRNA gene and sequencing of the purified PCR product were done as described previously [31]. The 16S rRNA gene sequences of the strains were then submitted to the EzBioCloud database (www.ezbiocloud.net/identify, accessed on 25 April 2025) [32] for similarity comparison analysis to determine their preliminary taxonomic position. The corresponding gene sequences of closely related species were retrieved from GenBank [33]. Multiple sequence alignment with the sequences of related type strains was performed using the Clustal W program implemented in MEGA software (version 12) [34]. Phylogenetic trees were reconstructed from the alignment using three independent algorithms: the Neighbor-Joining (NJ), Maximum-Likelihood (ML) and Maximum-Parsimony (MP) methods [35,36,37]. Evolutionary distances for the NJ and ML analyses were calculated based on the Kimura two-parameter model [38]. The robustness of the tree topology was evaluated by bootstrap analysis with 1000 replicates [39].

### 2.5. Whole-Genome Sequencing and Phylogenomic Analysis

The genomic DNA of strains YIM B11945^T^ and YIM B119997^T^ were extracted using an Invitrogen PureLink^®^ Genomic DNA kit and sequenced using the Pacific Biosciences Sequel II (PacBio) technology. The complete genome sequences were assembled and polished using unicycler [40] (https://github.com/rrwick/Unicycler, accessed on 1 September 2025), which combined both short reads and long reads. The genome sequences were analyzed for completeness and contamination rates using CheckM2 v1.0.1 [41]. Bakta version 1.7.0 [42] carried out annotation of the genomes of the strains. Additional whole-genome-based phylogenomic analysis of strains YIM B11945^T^ and YIM B11997^T^ was performed with the Type Strain Genome Server (TYGS) (https://tygs.dsmz.de) [43]. Digital DNA–DNA hybridization (dDDH) values were determined with the Genome-to-Genome Distance Calculator 3.0 (https://ggdc.dsmz.de/distcalc2.php, accessed on 6 September 2025, Formula: 2 (identities/HSP length)) [44]. The average nucleotide identity (ANI) was calculted via the EzBioCloud platform tool (https://www.ezbiocloud.net/tools/ani, accessed on 20 January 2026) [45].

The annotation of the genome was carried out using the Rapid Annotation using Subsystem Technology (RAST) web server version 2.0 [46,47]. In addition, AntiSMASH (https://antismash.secondarymetabolites.org/, accessed on 7 August 2026) was used to predict antibiotic resistance genes and secondary metabolite biosynthetic gene clusters (BGCs) in the strain genomes.

## 3. Results

### 3.1. Phylogenomic Analysis

**Figure 1:**
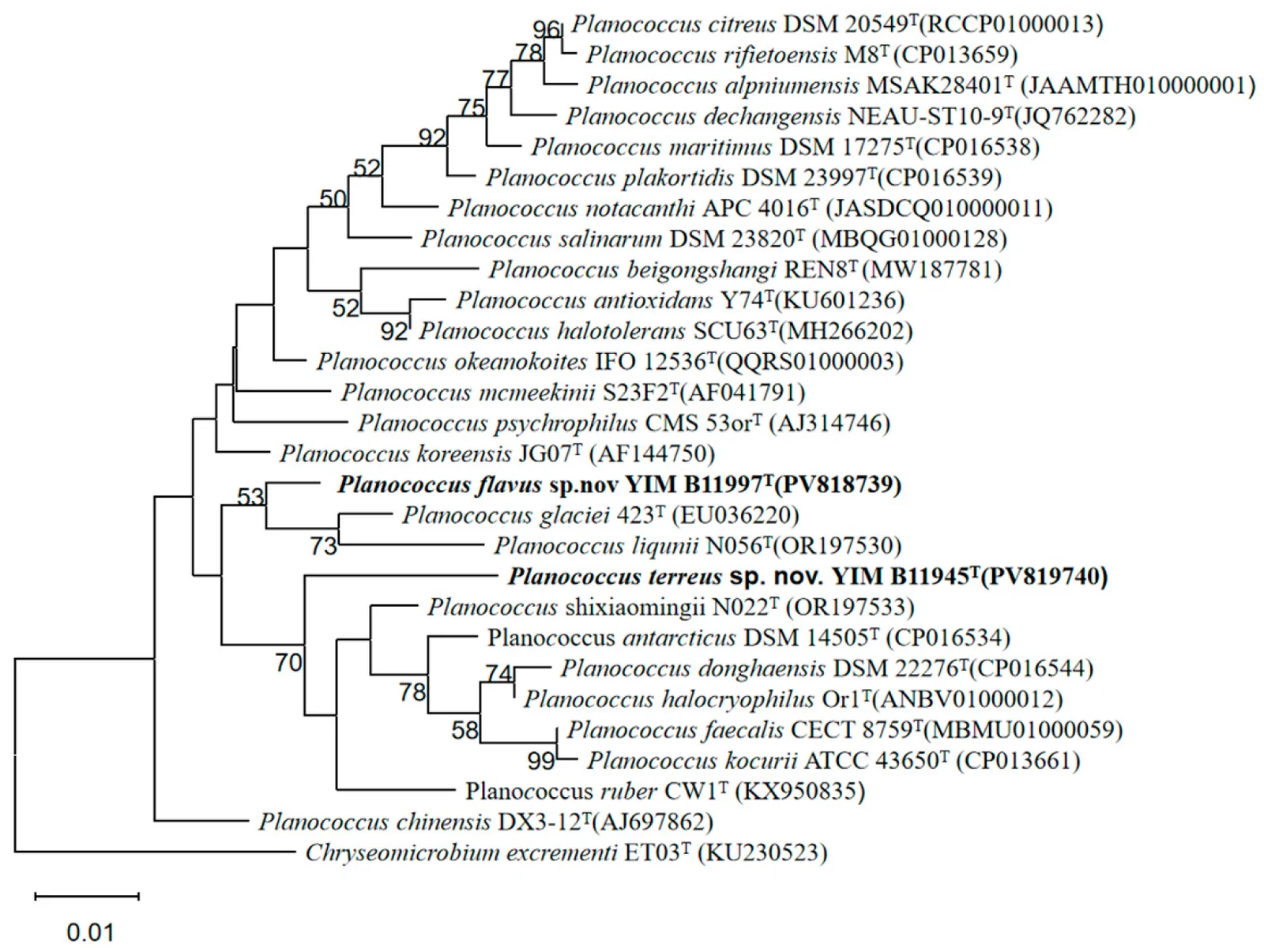
Maximum-Parsimony phylogenetic tree based on 16S rRNA gene sequences showing the relationships of the isolate and related taxa. Bootstrap values (expressed as percentages of 1000 replications) are shown at branching points; only bootstrap values of more than 50% are shown on the nodes. *Chryseomicrobium excrementi* ET03^T^ (KU230523) was used as the outgroup. Bar, 0.01 substitutions per nucleotide position.

**Figure 2:**
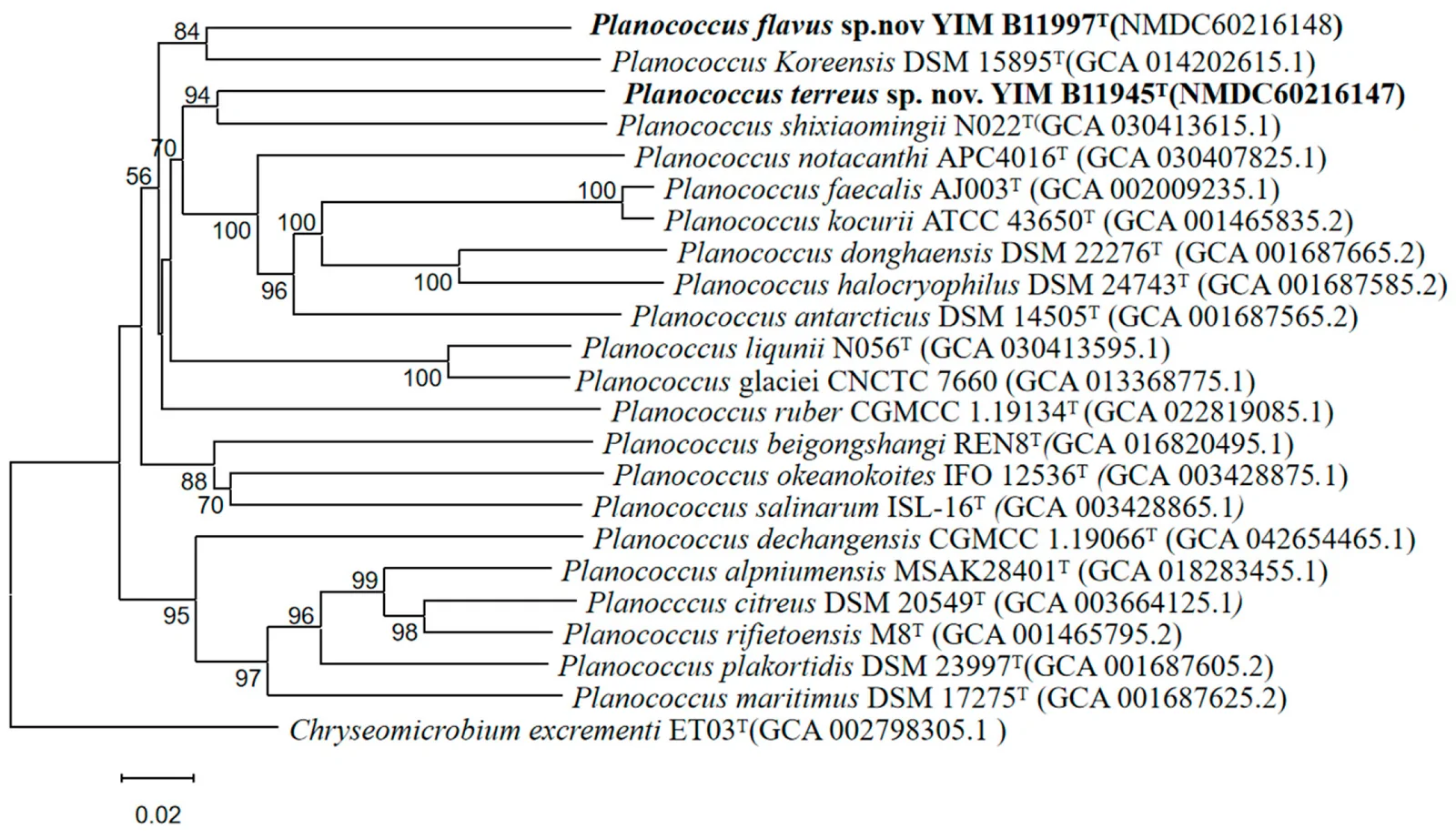
Whole-genome sequence tree generated with the TYGS web server for strains YIM B11945^T^ and YIM B11997^T^ and closely related species. Tree inferred with FastME 2.1.6.1 [45] from GBDP distances calculated from genome sequences. The branch lengths are scaled in terms of GBDP distance formula d5. The numbers above branches are GBDP pseudo-bootstrap support values >60% from 1000 replications. Bar, 0.02.

Nearly complete 16S rRNA gene sequences for strains YIM B11945^T^ (1418 bp) and YIM B11997^T^ (1423 bp) were obtained and deposited in the NCBI GenBank database under accession numbers PV818739 and PV818740, respectively. The sequence similarities between the PCR-amplified and genome-extracted sequences were 99.2% for YIM B11945^T^ and 100% for YIM B11997^T^, confirming their genomic authenticity. Homology searches for the 16S rRNA gene sequences were conducted using the EzBioCloud database to determine the closest phylogenetic relatives. The results showed that strain YIM B11945^T^ exhibited the highest sequence similarity with *Planococcus shixiaomingii* N022^T^ (97.5%), followed by *P. mcmeekinii* S23F2^T^ (97.2%) and *P. okeanokoites* IFO 12536^T^ (97.1%); strain YIM B11997^T^ showed the highest sequence similarity with *P. koreensis* JG07^T^ (98.3%), followed by *P. mcmeekinii* S23F2^T^ (98.2%) and *P. okeanokoites* IFO 12536^T^ (98.1%). The 16S rRNA gene-based phylogenetic Maximum-Likelihood tree (Figure 1) illustrates that the two strains cluster into distinct subclades in the genus *Planococcus*. There was a slight variability in the topologies of the Maximum-Parsimony and Neighbor-Joining trees (Figures S1 and S2; available in the online version of this article), likely attributable to the low bootstrap confidence. The phylogenomic tree further confirms that the strains YIM B11945^T^ and YIM B11997^T^ belong to the genus *Planococcus* and are most closely clustered with *P. shixiaomingii* N022^T^ and *P. koreensis* JG07^T^ (Figure 2), respectively.

### 3.2. Genome Features

The levels of completeness and contamination of the genomes were assessed using CheckM (version 1.0.18). The completeness and contamination levels of the genomic data of the two strains, YIM B11945^T^ and YIM B11997^T^, were 99.9%, 0.4% and 100%, 0.1%, respectively, demonstrating that the genome sequences determined were appropriate for later analysis. The chromosome of strain YIM B11945^T^ was 3,625,729 bp long, with a DNA G+C content of 45.0%. Gene prediction allowed the annotation of 3763 protein-coding sequences (CDSs), with 24 rRNA genes and 65 tRNA genes. The chromosome of strain YIM B11997^T^ was 3,707,144 bp, with a DNA G+C content of 49.5%. During gene prediction, strain YIM B11997^T^ was annotated with 3763 CDSs, 27 rRNA genes and 87 tRNA genes. The dDDH values between YIM B11945^T^ and *P. shixiaomingii* and between YIM B11945^T^ and YIM B11997^T^ were 22.5% and 20.7%, respectively. The dDDH value between YIM B11997^T^ and *P. koreensis* was 23.0%. The ANI values between YIM B11945^T^ and *P. shixiaomingii* and between YIM B11945^T^ and YIM B11997^T^ were 78.1% and 75.1%, respectively. The ANI value between YIM B11997^T^ and *P. koreensis* was 78.9%. The dDDH and ANI values of YIM B11945^T^ and YIM B11997^T^ and other close relatives of the genus are summarized in Table S1, and they are significantly below the cut-off values for species demarcation, suggesting that the strains YIM B11945^T^ and YIM B11997^T^ can be clearly distinguished from each other and their closely related type strains, representing two novel species within the genus *Planococcus*.

According to the RAST functional annotations, the most abundant subsystem categories included amino acids and derivatives, carbohydrates, cofactors/vitamins/prosthetic groups/pigments and protein metabolism (Figure S3). Furthermore, the genomes of strains YIM B11945^T^ and YIM B11997^T^ also revealed the presence of gene clusters for respiration, stress response, motility and chemotaxis. The presence of the gene cluster for motility and the presence of flagella indicated that the genomic and phenotypic results are consistent with each other. AntiSMASH predicted two terpene, one T3PKS and one terpene-precursor secondary metabolite cluster in strains YIM B11945^T^ and YIM B11997^T^. This result was similar to that predicted for strain *P. shixiaomingii* N022^T^, but it differed from the predictions for *P. koreensis* JG07^T^, and *P. okeanokoites* IFO 12536^T^.

### 3.3. Phenotypic Characteristics

The strains YIM B11945^T^ and YIM B11997^T^ were strictly aerobic, Gram-stain-positive, catalase- and oxidase-positive, short rod shaped, and motile with flagella (Figure S4). Both strains grew well on ISP2 and LB but grew slowly on TSA, R2A and NA media. After incubation for 3 days on ISP2, the colonies were observed to be round, convex, and smooth, but differing in that the colony color of strain YIM B11945^T^ was pink, whereas strain YIM B11997^T^ was light yellow. Strain YIM B11945^T^ was able to grow at 10–30 °C (optimally at 28 °C), and strain YIM B11997^T^ was able to grow at 20–30 °C (optimally at 28 °C). The growth of the two strains was observed in ISP2 broth with NaCl concentrations of 0–7% (w/v) at optimal temperatures. The optimum growth salt concentration of both strains was 0–1.0%. Strain YIM B11945^T^ could grow at pH 6.0–11.0 (optimum at pH 8.0) and strain YIM B11997^T^ could grow at pH 7.0–11.0 (optimum at pH 9.0) at the optimal temperature. Detailed phenotypic characteristics of these two novel strains are provided in Table 1, along with the species description.

Strain YIM B11945^T^ could be distinguished from its closely related phylogenetic neighbor, *P. shixiaomingii* N022^T^, by its specific growth conditions, ability to degrade gelatin and acid production capacity of D-glucose and melibiose. In contrast, strain YIM B11997^T^ could be differentiated from its close phylogenetic neighbor *P. koreensis* JG07^T^ by its acid production capacity of melibiose, as well as its distinct growth temperature and pH range (Table 1).

The chemotaxonomic analysis revealed that strains YIM B11945^T^ and YIM B11997^T^ exhibited characteristic features consistent with the genus *Planococcus*. Both strains contained diphosphatidylglycerol (DPG), phosphatidylglycerol (PG) and phosphatidylethanolamine (PE) as identical phospholipid profiles. Notably, one unidentified polar lipid (PL) and two unidentified PLs were additionally identified as the polar lipids in the strains YIM B11945^T^ and YIM B11997^T^, respectively (Figure S5). The fatty acid analysis demonstrated strain YIM B11945^T^ and its closely neighbor *P. shixiaomingii* N022^T^ [48] shared and strain-specific patterns (Table 2). The major fatty acids in both strains included antesio-C_15:0_ (43.9% and 40.4%) and C_16:1ω7c_ alcohol (10.5% and 14.9%). Strain-specific variation was observed, with YIM B11945^T^ showing elevated level of iso-C_17:1_ I and/or anteiso-C_17:1_ B (9.2%), antesio-C_17:0_ (6.1%), and iso-C_17:0ω10c_ (5.1%), while *P. shixiaomingii* N022^T^ contained iso-C_14:0_ (10.4%). Strain YIM B11997^T^ contained antesio-C_15:0_ (27.8%), iso-C_14:0_ (16.4%), C_16:1ω7c_ alcohol (16.1%) and iso-C_16:0_ (10.5%) as the major fatty acids, in line with those of *P. koreensis* JG07^T^ [8] (Table 2). Both strains contained menaquinone-8 and MK-7 as the primary isoprenoid quinones, which is consistent with the genus *Planococcus*.

## 4. Conclusions

Based on the phylogenetic, phylogenomic, phenotypic, and chemotaxonomic analyses, as well as the genome comparisons, strains YIM B11945^T^ and YIM B11997^T^ are proposed to represent two novel species within the genus *Planococcus*, for which the name *Planococcus terreus* sp. nov. and *Planococcus flavus* sp. nov. are proposed, respectively.

### 4.1. Description of Planococcus terreus sp. nov.

The cells are Gram-stain-positive, aerobic, and motile by means of flagella. The cells measure 0.4 to 0.5 μm in width and 0.6 to 0.7 μm in length. The colonies are 1–3 mm in diameter, round, convex, smooth, and pink-orange after 2–3 days of incubation at 28 °C on ISP2 plates. Growth occurs at 10–30 °C (optimum, 28 °C), pH 6.0–11.0 (optimum, pH 8.0), and 0–7% (w/v) NaCl (optimum, 0–1%). The cells are positive for oxidase and catalase. The organism is found to hydrolyze esculin, gelatin, and Tween 20, but not starch, urea, or Tween 40 and 80. it is positive for alkaline phosphatase, esterase (C4), esterase lipase (C8), leucine arylamidase, valine arylamidase, cystine arylamidase, trypsin, a-chymotrypsin, acid phosphatase, naphthol-AS-BI-phosphohydrolase and β-glucosidase, and negative for the reduction of nitrates, indole production, H_2_S production, oxidation and fermentation of glucose, urease, arginine dihydrolase, lipase (C14), a-galactosidase, β-galactosidase, β-glucuronidase, a-glucosidase, N-acetyl-β-glucosaminidase, a-mannosidase and a-fucosidase. Acids are produced from D-ribose, D-xylose, D-glucose, D-fructose, D-mannitol, N-acetylglucosamine, D-maltose, and D-trehalose. It utilizes the following carbohydrates as the sole carbon sources: D-maltose, gentiobiose, D-salicin, D-trehalose, D-glucose N-acetyl-D-glucosamine, D-fructose, D-glucose-6-phosphate, L-alanine, D-gluconic acid, α-hydroxy-butyric acid and acetic acid. The dominant quinones are MK-8 and MK-7. The polar lipids are PG, PE, DPG and PL. The major cellular fatty acids are antesio-C_15:0_, C_16:1ω7c_ alcohol, summed feature 4 (iso-C_17:1_ and/or anteiso-C_17:1_B), iso-C_15:0_, antesio-C_17:0_, iso-C_17:1ω10_, and iso-C_16:0_. The whole genome assembly comprises 3.6 Mbp and exhibits a DNA G+C content of 45.0%.

The type strain YIM B11945^T^=CGMCC 1.64737^T^ =KCTC 43683^T^) was isolated from a soil sample collected from the Napahai Plateau wetland, P.R. China. The GenBank accession no. for the 16S rRNA gene sequence reported in this paper is PV818739. The genome sequence has been deposited at the National Microbiology Data Center under the accession no. NMDC60216147.

### 4.2. Description of Planococcus flavus sp. nov.

The cells are Gram-stain-positive, aerobic, and motile by means of flagella. The cells measure 0.6–0.7 μm in width 1.0–1.3 μm in length. The colonies are 1–3 mm in diameter, round, convex, smooth, and light yellow after 2–3 days of incubation at 28 °C on ISP2 plates. Growth occurs at 10–30 °C (optimum, 28 °C), pH 7.0–11.0 (optimum, pH 9.0), and 0–7% (w/v) NaCl (optimum, 0–1%). The organism is positive for oxidase and catalase. It is able to hydrolyze esculin gelatin and Tween 20, but not starch, urea, or Tween 40 and 80. it is positive for alkaline phosphatase, esterase (C4), esterase lipase (C8), leucine arylamidase, valine arylamidase, cystine arylamidase, trypsin, α-chymotrypsin, naphthol-AS-BI-phosphohydrolase, α-glucosidase and β-glucosidase, and it is negative for the reduction of nitrates, indole production, H_2_S production, oxidation and fermentation of glucose, urease, arginine dihydrolase, lipase (C14), acid phosphatase, α-galactosidase, β-galactosidase, β-glucuronidase, N-acetyl-β-glucosaminidase, α-mannosidase and α-fucosidase. Acids are produced from aesculin ferric citrate and potassium 5-ketogluconate. It utilizes the following carbohydrates as the only carbon sources: D-fructose and L-alanine. The dominant quinones are MK-8 and MK-7. The polar lipids are DPG, PE, PG, PL1 and PL2. The major cellular fatty acids are antesio-C_15:0_, iso-C_14:0_, C_16:1ω7c_ alcohol, iso-C_16:0_, and C_16:0_. The whole genome assembly comprises 3.7 Mbp and exhibits a DNA G+C content of 49.5%.

The type strain YIM B11997^T^=KCTC 43680^T^=NBRC 116630^T^) was isolated from a soil sample collected from the Napahai Plateau wetland, P.R. China. The GenBank accession no. for the 16S rRNA gene sequence reported in this paper is PV818740. The genome sequence has been deposited at DDBJ/ENA/GenBank under the accession no. NMDC60216148.

## Figures and Tables

**Table 1:** Differential characteristics of strains YIM B11945^T^ and YIM B11997^T^ and their close relatives.

Characteristic	1	2	3	4	5	6
Colony color	Pink-orange	Light orange	Pale yellow	Yellow-orange	Bright yellow/orange	Pale orange
Cell shape	Short rods	Rods	Short rods	Cocci or short rods/rods	ND	Cocci or short rods
Temperature range for growth (°C)	10–30	4–42	20–30	4–39	20–37	10–37
Temperature optimum (°C)	28	20	28	20–30	ND	ND
NaCl range for growth (%, w/v)	0–7	0–11	0–7	0–7	0–7	0–14
NaCl optimum (%, w/v)	0–1	2–4	0–1	1–4	1–4	ND
pH range for growth	6–11	7–10	7–11	5.5–10	ND	ND
pH optimum	8	9	9	7.0–8.5	ND	ND
Oxidase	+	–	+	–	W	–
Nitrate reduction	–	–	–	–	–	+
Hydrolysis of						
Gelatin	+	–	+	+	+	+
Aesculin	+	+	+	+	–	–
Acid production from:						
D-glucose	+	–	–	W	–	+
Fructose	+	+	–	–	+	+
D-Mannitol	+	+	–	–	–	–
Aesculin ferric citrate	–	+	+	+	–	–
Potassium 5-ketogluconate	–	+	+	+	+	+
Melibiose	–	+	–	+	–	–
Predominant menaquinones	MK-7, MK-8	MK-7, MK-8	MK-7,MK-8	MK-7, MK-8, MK-6	MK-7, MK-8	ND
G+C (%)	45.0	43.5	49.6	47.0	46.3	35.0

Strains: 1, YIM B11945^T^; 2, *Planococcus shixiaomingii* N022^T^ [48]; 3, YIM B11997^T^; 4, *P. koreensis* JG07^T^ [3]; 5, *P. okeanokoites* IFO 12536^T^ [3,13,49]; 6, *P. mcmeekinii* S23F2^T^ [7,48]. “+”: positive, “–”: negative, “W”: weakly positive, “ND”: not determined.

**Table 2:** Comparison of the fatty acid (%) profiles of strains YIM B11945T and YIM B11997T and type strains of closely related species in the genus *Planococcus*.

Fatty Acid	1	2	3	4	5	6
C_15:0_	-	-	**-**	2.4	**-**	1.6
C_16:0_	2.1	**-**	5.9	**-**	1.1	1.1
C_16:0_	-	-	1.1	**-**	-	-
C_18:0_	TR	-	3.0	-	-	-
Iso-C_14:0_	3.6	**10.4**	**16.4**	**21.0**	**40.4**	**18.1**
Iso-C_15:0_	6.1	3.5	4.3	6.4	-	5.3
Anteiso-C_15:0_	**43.9**	**40.4**	**27.8**	**26.1**	4.0	**32.0**
Iso-C_16:0_	5.3	9.9	**10.5**	**11.1**	**24.5**	8.7
Iso-C_17:0_	3.9	-	1.5	1.6	-	1.3
Anteiso-C_17:0_	6.1	3.0	2.0	1.6	-	1.2
C_16:1_ω7c alcohol	**10.5**	**14.9**	**16.1**	**23.6**	**26.1**	**22.9**
C_16:1_ω11c	2.0	4.7	1.8	1.7	2.1	2.3
Iso-C_17:1_ω5c	-	-	-	2.8	-	-
Iso-C_17:1_ω10c	5.9	-	1.8	1.9	-	2.4
C_18:1_ω9c	NR	-	-	-	1.0	-
Summed feature 4 *	9.2	-	3.7	-	3.0	-

Strains: 1, YIM B11945^T^; 2, *P. shixiaomingii* N022^T^; 3, YIM B11997^T^; 4, *P. koreensis* JG07^T^; 5, *P. okeanokoites* IFO 12536^T^; 6, *P. mcmeekinii* S23F2^T^. Bold type indicates the major fatty acids (>10.0%); TR, trace amount (<1.0%); −, not detected. Data for strains 1 and 3 were taken from this study, data for strain 2 was taken from reference [49], data for strains 4–6 were taken from reference [8]. * Summed feature 4 represents iso-C17:1 I and/or anteiso-C17:1 B, which could not be separated by GLC with the MIDI system.

## Data Availability

The 16S rRNA gene and genome sequences of strains YIM B11945^T^ and YIM B11997^T^ have been deposited in the GenBank/EMBL/DDBJ database and the National Microbiology Data Center (NMDC) under the accession numbers PV818739 and PV818740 and NMDC60216147 and NMDC60216148, respectively.

## Supplementary Materials

The following supporting information can be downloaded at: https://www.mdpi.com/article/10.3390/microorganisms14092119/s1, Figure S1: Neighbor-Joining phylogenetic tree based on 16S rRNA gene sequences showing the relationships of the isolate and related taxa. Bootstrap values (expressed as percentages of 1000 replications) are shown at branching points; only bootstrap values with more than 50% are shown on the nodes. *Chryseomicrobium excrementi* ET03^T^ (KU230523) was used as the outgroup. Bar, 0.01 substitutions per nucleotide position; Figure S2: Maximum-Parsimony phylogenetic tree based on 16S rRNA gene sequences showing the relationships of the isolate and related taxa. Bootstrap values (expressed as percentages of 1000 replications) are shown at branching points; only bootstrap values with more than 50% are shown on the nodes. *Chryseomicrobium excrementi* ET03^T^ (KU230523) was used as the outgroup; Figure S3: RAST subsystem annotation of the genome of strains YIM B11945^T^ (A) and YIM B11997^T^ (B). The figures shows subsystem coverage, subsystem category distribution and subsystem feature counts; Figure S4: Transmission electron microscope images of strains YIM B11945^T^ (A) and YIM B11997^T^ (B) after 3 days of growth on ISP2 agar at 25 °C. Bar, 0.5 μm; Figure S5: Polar lipids of strains YIM B11945^T^ (A) and YIM B11997^T^ (B) after separation by two dimensional TLC. The polar lipids detected were DPG, diphosphatidylglycerol; PG, phosphatidylglycerol; PE, phosphatidylethanolamine; and PL, unidentified polar lipid; Table S1: The pairwise dDDH (upper right triangle) and ANI (lower right triangle) values (%) between the two strains and their closest relatives.

## Abbreviations

The following abbreviations are used in this manuscript:

ANI	Average nucleotide identity
dDDH	Digital DNA–DNA hybridization
LB	Luria–Bertani
TSA	Tryptic soy agar
R2A	Reasoner’s 2A
NA	Nutrient agar
ISP2	International Streptomyces Project 2
NJ	Neighbor-Joining
ML	Maximum-Likelihood
MP	Maximum-Parsimony
DPG	Diphosphatidylglycerol
PG	Phosphatidylglycerol
PE	Phosphatidylethanolamine
PL	Unidentified polar lipid
