# Supplementary material for: Planococcus terreus sp. nov. and Planococcus flavus sp. nov., Two New Species Isolated from Soil of the Napahai Plateau Wetland
Source: Microorganisms. 2026 Sep 21;14(9):2119. doi: 10.3390/microorganisms14092119 (PMC13609399; doi:10.3390/microorganisms14092119)
Supplement: Supplementary file 1 [file microorganisms-14-02119-s001.zip › microorganisms-4574047-supplementary.pdf]

Planococcus terreus sp. nov. and *Planococcus flavus* sp. nov. two new species isolated from soil of the Napahai Plateau wetland

Weidong Lu[1], Xingfeng Qian[1], Zhen Zhang[1], Zhiying Li[1], Zefen Yu[2], Yunxia Tian[3], Li Li[4*], Yongxia Wang[1*]

1Yunnan Institute of Microbiology, School of Life Sciences, Yunnan University, Kunming, 650091, P.R. China
2State Key Laboratory for Conservation and Utilization of Bio-Resources in Yunnan, Yunnan University, Kunming 650504, China
3Kunming Academy of Agricultural Sciences, Kunming, 650034, P.R. China
4Xinjiang Key Laboratory of Biodiversity Conservation and Application in Arid Lands, Xinjiang Institute of Ecology and Geography, Chinese Academy of Sciences, Urumqi 830011, China

* Corresponding authors: Li Li (lili.bobo@outlook.com); Yongxia Wang (wangyx@ynu.edu.cn)

Table S1. The pairwise dDDH (upper right triangle) and ANI (lower right triangle) values (%) between the two strains and their closest relatives.

Strains: 1, YIM B11945^T; 2, *P. shixiaomingii* N022^T; 3, YIM B11997^T; 4, *P. koreensis* JG07^T; 5, *P. okeanokoites* IFO 12536^T

	1	2	3	4	5
1	-	22.5	20.7	20.1	19.5
2	78.1	-	21.0	20.3	19.6
3	75.1	74.8	-	23.0	19.8
4	74.7	74.8	78.9	-	19.5
5	74.4	73.9	74.3	74.5	-

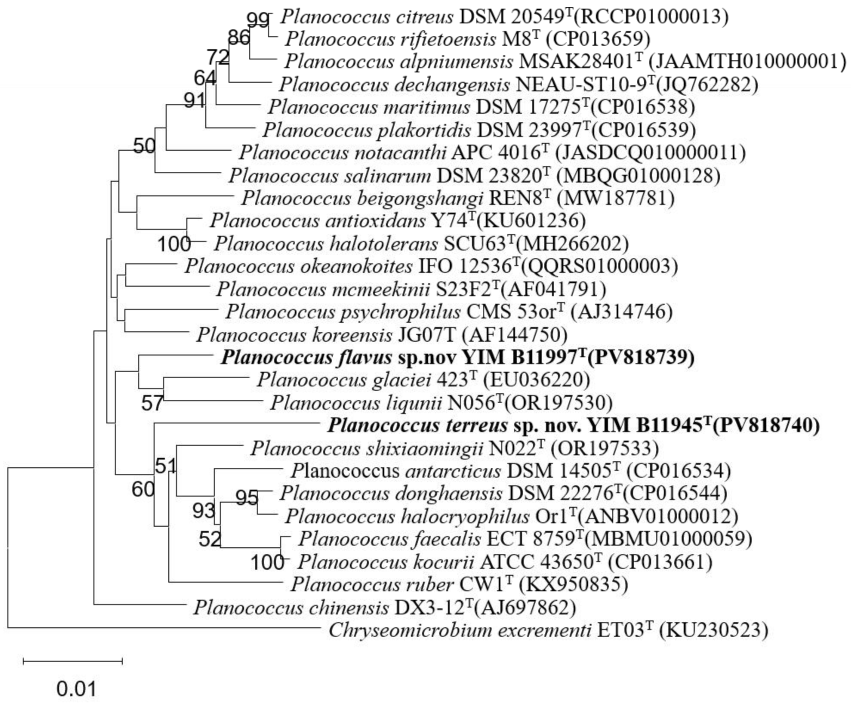

Figure S1. Neighbour-joining phylogenetic tree based on 16S rRNA gene sequences showing the relationships of the isolate and related taxa. Bootstrap values (expressed as percentages of 1000 replications) are shown at branching points; only bootstrap values with more than 50 % are shown on the nodes. *Chryseomicrobium excrementi* ET03^T (KU230523) was used as the outgroup. Bar, 0.01 substitutions per nucleotide position.

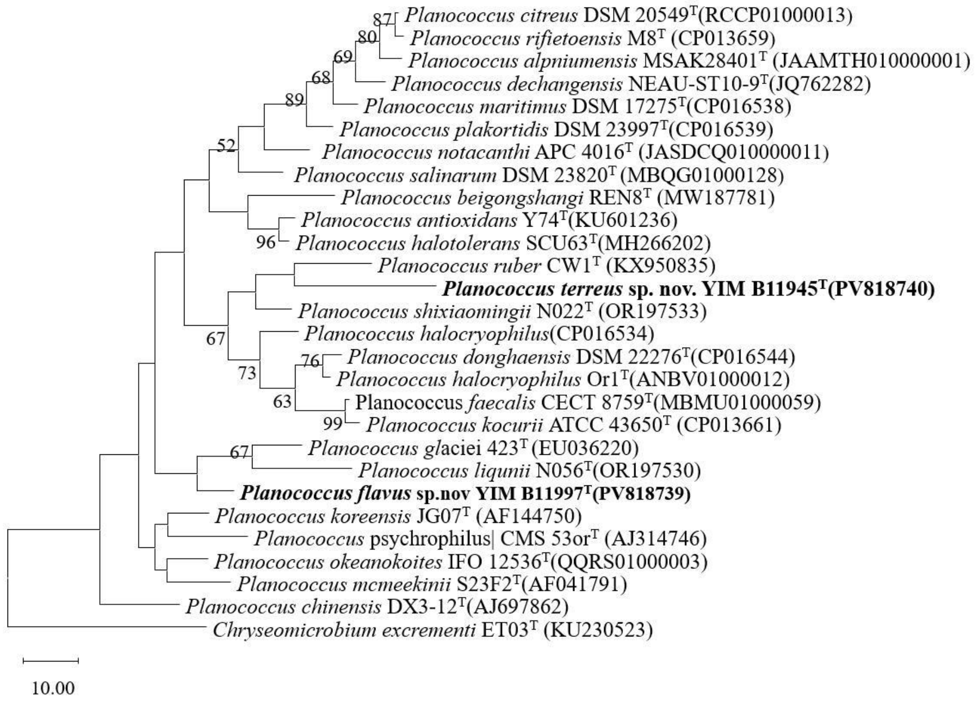

Figure S2. Maximum-parsimony phylogenetic tree based on 16S rRNA gene sequences showing the relationships of the isolate and related taxa. Bootstrap values(expressed as percentges of 1000 replications) are shown at branching points; only bootstrap values with more than 50 % are shown on the nodes. *Chryseomicrobium excrementi* ET03^{T} (KU230523) was used as the outgroup.

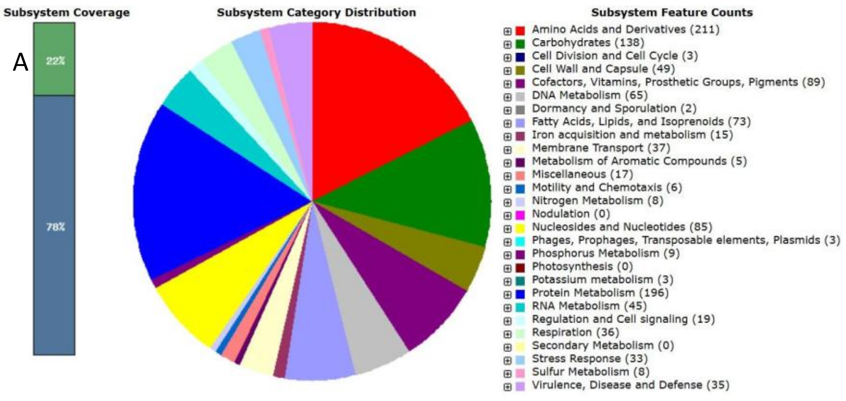

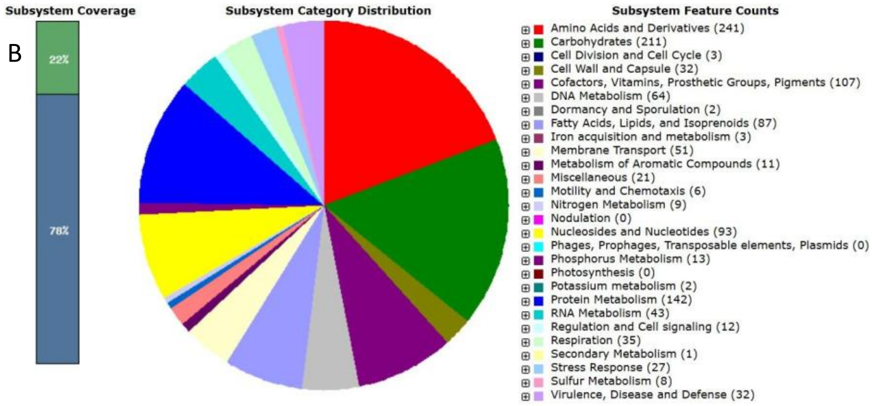

Figure S3. RAST subsystem annotation of the genome of strains YIM B11945^T (A) and YIM B11997^T(B). The figures shows subsystem coverage, subsystem category distribution and subsystem feature counts.

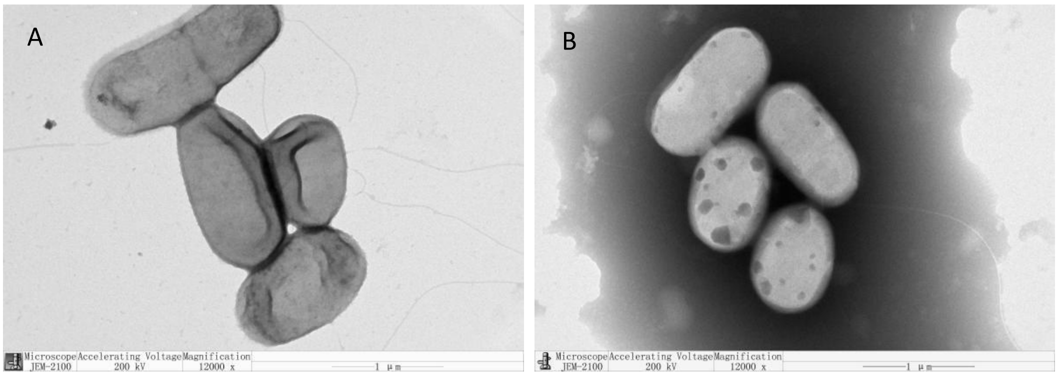

Figure S4. Transmission electron microscope images of the **c**ell morphology of strains YIM B11945^T (A) and YIM B11997^T (B), after 3 days growth on ISP2 agar at 25 °C. Bar,1 μm.

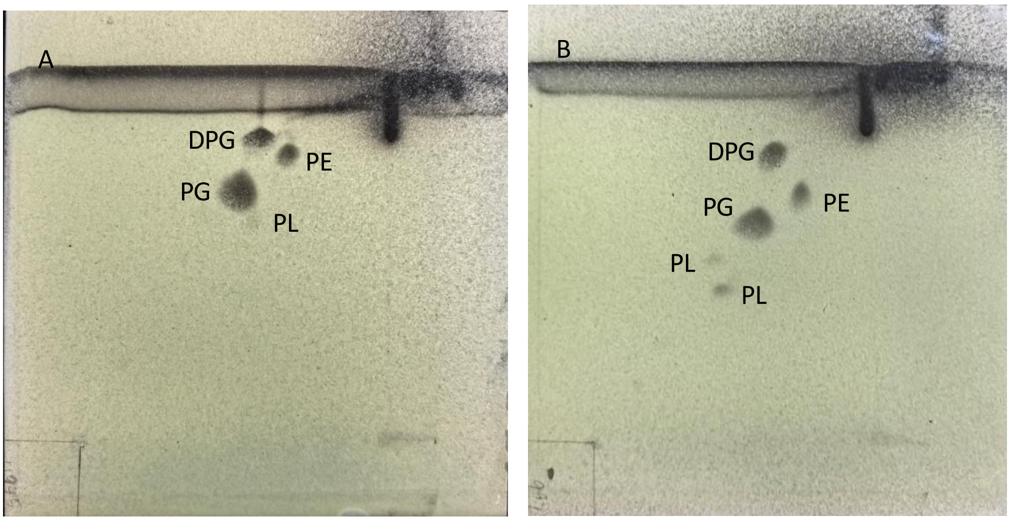

Figure S5. Polar lipids of strains YIM B11945^T (A) and YIM B11997^T (B) after separation by two dimensional TLC.The polar lipids detected were DPG, diphosphatidylglycerol; PG,phosphatidylglycerol; PE, phosphatidylethanolamine; and PL, unidentified polar lipid.
